# *secA*,* secD*,* secF*,* yajC,* and *yidC* contribute to the adhesion regulation of *Vibrio alginolyticus*

**DOI:** 10.1002/mbo3.551

**Published:** 2017-10-23

**Authors:** Lina Guo, Lixing Huang, Yongquan Su, Yingxue Qin, Lingmin Zhao, Qingpi Yan

**Affiliations:** ^1^ Fisheries College Key Laboratory of Healthy Mariculture for the East China Sea Ministry of Agriculture Jimei University Xiamen, Fujian China; ^2^ State Key Laboratory of Large Yellow Croaker Breeding Ningde Fujian China; ^3^ College of Ocean & Earth Sciences Xiamen University Xiamen Fujian China

**Keywords:** adhesion, environmental factors, RNAi, *secA*, *secD*, *secF*, *Vibrio alginolyticus*, *yajC*, *yidC*

## Abstract

*Vibrio alginolyticus* caused great losses to aquaculture. Adhesion is an important virulence factor of *V. alginolyticus*. In this study, the relationship between *V. alginolyticus* adhesion and type II secretion system genes (*secA*,* secD*,* secF*,* yajC,* and *yidC*) was determined using gene silencing, qRT‐PCR and in vitro adhesion assay. The results showed that the expression of target genes and the bacterial adhesion exhibited significant decreases after transient gene silencing and stable gene silencing, which indicated that *secA*,* secD*,* secF*,* yajC,* and *yidC* played roles in the bacterial adhesion of *V. alginolyticus*. The expression of *secA*,* secD*,* secF*,* yajC,* and *yidC* were significantly influenced by temperature, salinity, pH and starvation. The results indicated that the expression of *secA*,* secD*,* secF*,* yajC,* and *yidC* were sensitive to different environmental factors, whereas environmental factors can affect *V. alginolyticus* adhesion via the expression of *secA*,* secD*,* secF*,* yajC,* and *yidC*.

## 1 INTRODUCTION

*Vibrio alginolyticus* is a gram‐negative halophilic bacterium that is distributed worldwide. *V. alginolyticus* is often associated with severe infection in maricultured animals and cause heavy economy losses (Luo et al., 2016; Yan, Chen, Ma, Zhuang, & Wang, 2007). In view of its perniciousness, the pathogenicity of *V. alginolyticus* has been intensively studied recently. Adhesion of pathogens to the surface of hosts is considered as an important initial step of infection process (Chen, Yan, Wang, Zhuang, & Wang, 2008). Although several genes have been demonstrated to be involved in the adhesion of *V. alginolyticus* (Huang et al., 2015, Huang, Hu, et al., 2016a, 2017; Luo et al., 2016; Wang et al., 2015), the molecular mechanisms of *V. alginolyticus* adhesion still call for further investigation.

To better understand the regulation of *V. alginolyticus* adhesion, *V. alginolyticus* was cultured under nine kinds of stress conditions, respectively. Our results showed that, compared with the unstressed control, exposure of *V. alginolyticus* to Cu^2+^, Pb^2+^, Hg^2+^ and low pH resulted in significant reduction in adhesion (Kong et al., 2015). RNA‐Seq showed that *secA*,* secD*,* secF*,* yajC,* and *yidC*, which belong to the type II secretion system, were down regulated under stresses of Cu^2+^, Pb^2+^, Hg^2+^, and low pH (the data have been deposited in the NCBI Sequence Read Archive (SRA) under accession number SRP049226). These indicated that type II secretion system might play roles in the regulation of *V. alginolyticus* adhesion.

Gram‐negative bacteria possess a number of sophisticated secretion systems to transport virulence factors across the cell envelope, including the type II secretion system (T2SS) (Johnson, Waack, Smith, Mobley, & Sandkvist, 2016; Rule et al., 2016). One common way through which bacteria mediate pathogenesis is the secretion of proteins or toxins (Harding, Kinsella, Palmer, Skaar, & Feldman, 2016). It was found that the type II secretion system can secrete lipase and protease (Harding et al., 2016). SecA is an ATPase that provides energy to transfer a precursor polypeptide (Suo, Hardy, & Randall, 2015). SecYEG interacts with the motor protein SecA to mediate the translocation of secretory proteins (Prabudiansyah, Kusters, Caforio, & Driessen, 2015). *secD/F* mutant strain exhibits both severe cold sensitivity and a Sec‐specific protein translocation defect. SecD and SecF are closely related to the translocation machineries (Hand, Klein, Laskewitz, & Pohlschröder, 2006). SecD and SecF possess a very large first periplasmic domain that is important for catalyzing protein translocation (Nouwen, Piwowarek, Berrelkamp, & Driessen, 2005). Cells lacking SecD and/or SecF have a severe export defect and are barely viable (Pogliano & Beckwith, 1994a, 1994b). YidC provides new mechanistic insights of how transmembrane proteins achieve the transition from an aqueous environment in the cytoplasm to the hydrophobic lipid bilayer environment of the membrane (Kuhn & Kiefer, 2016). The *yajC* gene encodes the smaller subunit of the preprotein translocase complex, which interacts with membrane protein SecD and SecF to coordinate protein transport and secretion across cytoplasmic membrane. The YajC protein was linked to sensitivity to growth temperatures in *E. coli*, involved in translocation of virulence factors during Listeria infection, and stimulating a T‐cell‐mediated response of Brucella abortus (Liu, Skory, Qureshi, & Hughes, 2016).

For a better understanding of the role of type II secretion in the bacterial adhesion, the *secA*,* secD*,* secF*, yajC, and *yidC* genes were knocked down by RNAi, and then the adhesion ability were tested in this study.

## 2 MATERIALS AND METHODS

### 2.1 Bacterial strains and culture conditions

Pathogenic *V. alginolyticus* (ND‐01) was isolated from naturally infected *Pseudosciaene crocea* and previously identified as pathogenic by subsequent artificial infection (Yan et al., 2001; Kong et al., 2015). Bacteria were preserved in physiological saline with 10% glycerol at −80°C. *V. alginolyticus* was maintained at 28°C on tryptic soy agar (TSA) and grown in Luria–Bertani (LB) broth. Both of the culture mediums were supplemented with 2% NaCl to facilitate the bacterial growth.

For the investigation of effects of different temperatures, *V. alginolyticus* was prepared as below. Bacteria were incubated overnight in LB broth at 4°C, 15°C, 28°C, 37°C, and 44°C, respectively. Six replicates were performed for each treatment. After harvesting and resuspending, the bacterial suspensions were equilibrated at the same temperature for 30 min.

For the investigation of effects of different NaCl concentrations, *V. alginolyticus* were incubated overnight in LB broth with different NaCl concentrations (0.5, 1.5, 2.5, 3.5, and 4.5%). The bacterial cultures were washed with phosphate‐buffered saline (PBS) with different NaCl concentrations (0.5, 1.5, 2.5, 3.5, and 4.5%).

For the investigation of effects of different pH values, *V. alginolyticus* was treated as the following steps. Bacteria were incubated overnight in LB broth with different pH values (pH5, 6, 7, 8, and 9). The bacterial cultures were washed with phosphate‐buffered saline (PBS) with different pH values (pH 5, 6, 7, 8, and 9).

For the evaluation of influence of starvation, *V. alginolyticus* was prepared as below. *V. alginolyticus* was suspended in PBS, and the bacterial suspensions were adjusted to OD_560 _= 0.3, and then starved at 28°C for 1, 3, 5, and 7 day, respectively. Culturable *V. alginolyticus* cells were counted using plate counting.

All the above bacterial suspensions were adjusted to OD_560 _= 0.3 for RNA extraction, followed by qRT‐PCR. The counterpart bacterial suspensions were subjected to in vitro adhesion assays. Six replicates were performed for each treatment.

*E. coli* strain SM10 was obtained from TransGen Biotech (Beijing, China) and incubated in LB broth or on LB agar plates at 37°C.

### 2.2 Transient gene silencing

Short interfering RNA (siRNA) was designed base on gene sequences and synthesized by GenePharma Co., Ltd. (Shanghai, China). The treatment siRNA sequences and negative control were listed in Table S1.

Electroporation was performed according to the following method described by Huang et al. (2015). 2 μl siRNA (20 μmol/L) was added to a 100‐μl volume of competent cells, and stored on ice for 30 min, then the mixture were transferred to a cuvette, and immediately subjected to electroporation (1.8 KV, 6 ms). Then, 900 μl LB medium was added, and the mixture was incubated for 1, 6, 12, and 24 hr at 28°C prior to RNA extraction and qRT‐PCR.

### 2.3 Stable gene silencing

Stable gene silencing was performed according to the following method described by Luo et al. (2016). Five short hairpin RNA sequences targeting the coding regions of *secA*,* secF*,* yajC*,* secD,* and *yidC* mRNAs were synthesized by Shanghai Generay Biotech Co., Ltd. (Shanghai, China) (Table S2). After ligating the annealed oligonucleotides to the pACYC184 vector double digested with *Bam*HI and *Sph*I, the recombinant plasmids were transformed into *E. coli* SM10 via heat shock. The recombinant plasmids were further transferred from strain SM10 to *V. alginolyticus* by conjugation. An empty pACYC184 vector was used as the control. The stable silenced clones were screened by chloramphenicol (34 μg/ml).

### 2.4 Rna extraction and reverse transcription

TRIzol (Invitrogen, Carlsbad, CA, USA) was used to extract the bacterial total RNA according to the manufacturer's recommended protocol. A Revert Aid Mu‐MLV cDNA synthesis kit (Invitrogen) was used to synthesize the first‐strand cDNA from 2 mg of total RNA according to the manufacturer's recommended protocol.

### 2.5 Quantitative real‐time PCR (qRT‐PCR)

Power SYBR Green PCR Master Mix (Applied Biosystems, Carlsbad, California, USA) was used to assay the expression of genes by qRT‐PCR according to the manufacturer's instructions. 16s RNA was used to normalize the expression levels. The Relative Expression Software Tool (REST 2008.‐version 2) was used to calculate the relative expression of genes in RT‐qPCR using the Pair Wise Fixed Reallocation Randomization Test (Pfaffl, Horgan, & Dempfle, 2002). The mathematical model used was based on the mean crossing point deviation between the sample and the control group, normalized by the mean crossing point deviation of the reference genes. Specific amplification efficiencies were included in the correction of the quantification ratio. Significant differences between groups were determined by ANOVA followed by the Tukey's LSD. The primers are listed in Table S3.

### 2.6 Mucus preparation

Fifty individuals of healthy *Pseudosciaene crocea* were obtained from Ningde of Fujian Province, China. Skin mucus was prepared following the method of Yan et al. (2007). Fish surface was washed with sterile PBS (0.01 mol/L, pH 7.2), and then a plastic spatula was used to scrape the surface gel layer of the skin. This layer was homogenized in PBS, and the homogenate was centrifuged twice (20,000*g*, 4°C, 30 min) to remove particulate materials and then filtered through 0.45‐ and 0.22‐μm pore size filters, respectively. The mucus samples were adjusted to 1 mg protein/ml.

### 2.7 In vitro adhesion assay

The bacterial adhesion was determined according to the following method described by Huang, Huang, et al. (2016b). After spreading 50 μl of mucus evenly onto a 22 × 22 mm glass slide area, the mucus was fixed with methanol for 20 min. After adding 1 ml of bacterial suspension (10^8^ CFU/ml) onto the mucus‐coated glass slides, the slides were incubated at 25°C for 2 hr in a humidified chamber, followed by washing five times with PBS to remove nonadhering bacterial cells. Finally, the adhering bacterial cells were fixed with 4% methanol for 30 min, followed by dying with crystal violet for 3 min, and observed under a microscope (×1,000). The average number of bacteria adhering to a field of view of the glass surface was then determined. For each assay, 20 fields of view were counted and the average value was calculated. PBS without bacteria was used as the negative control.

### 2.8 Data processing

The data are presented as mean ± *SD*. Statistical analysis was performed by one‐way analysis of variance with Dunnett's test using the SPSS 13.0 software (Chicago, IL, USA). A value of *p *<* *.05 indicated a significant difference.

## 3 RESULTS

### 3.1 Validation for RNA‐Seq

**Figure 1:**
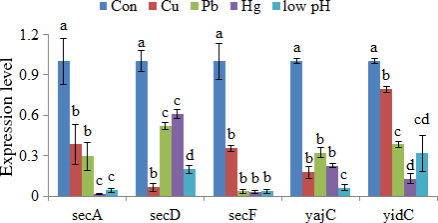
qRT‐PCR analysis of the expression of five genes after stress treatments compared with untreated control. The data are presented as the means ± *SD*, each treatment consisted of six independent biological replicates (3 technical replicates within each). The means of treatments not sharing a common letter are significantly different at *p *<* *.05

To verify the results obtained by RNA‐Seq, qRT‐PCR was performed on the five genes. The qRT‐PCR results were consistent with those in the RNA‐Seq results. The Cu^2+^, Pb^2+^, Hg^2+^, and low‐pH treatments significantly downregulated the expression of *secA* (by 2.61‐, 3.40‐, 58.82‐, and 23.36‐fold, respectively), *secD* (by 15.33‐, 1.92‐, 1.65‐, and 5.00‐fold, respectively), *secF* (by 2.82‐, 28.57‐, 33.33‐, and 27.78‐fold, respectively), *yajC* (by 5.61‐, 3.11‐, 4.46‐, and 16.13‐fold, respectively), and *yidc* (by 1.26‐, 2.61‐, 7.71‐, and 3.14‐fold, respectively) (Figure 1). These results further reinforced the reliability of the RNA‐Seq data in this study.

### 3.2 Effects of transient gene silencing

**Figure 2:**
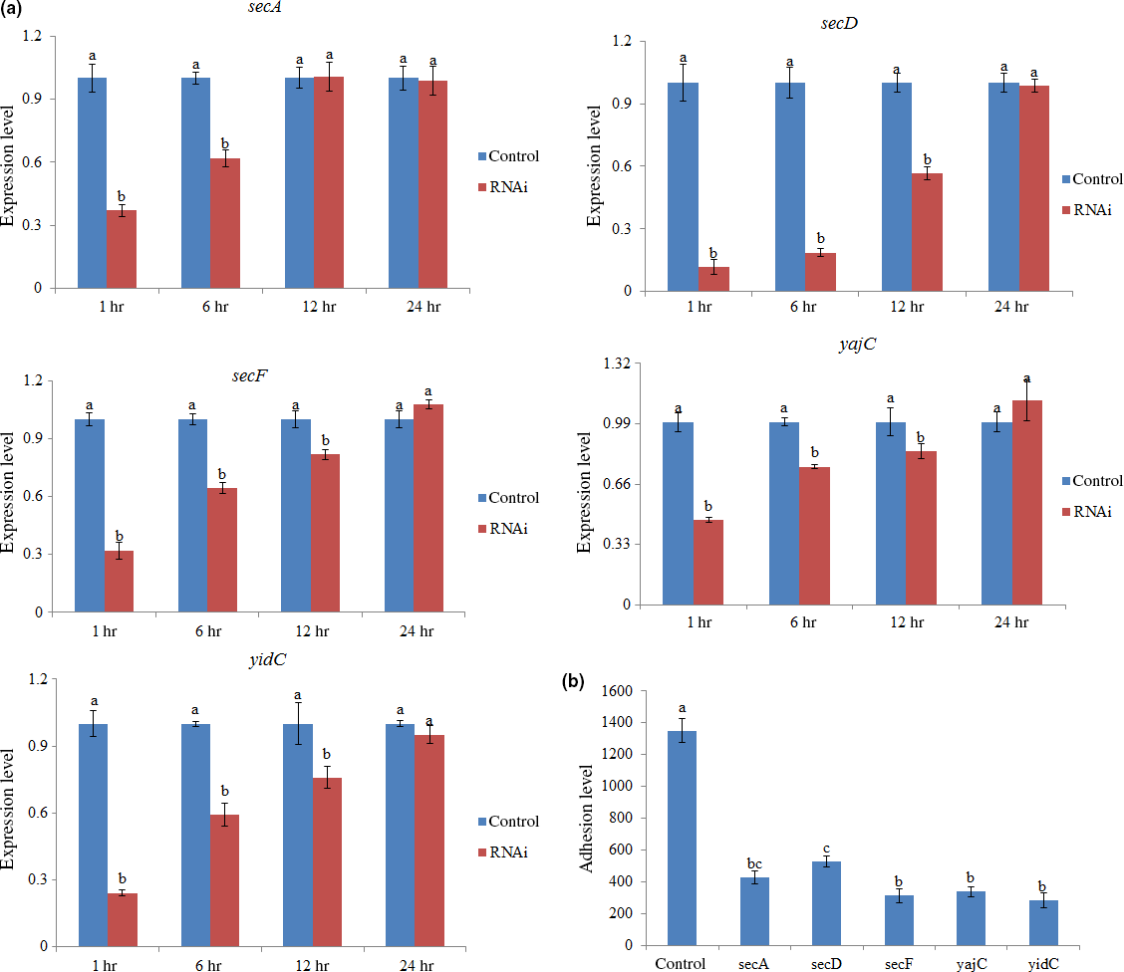
Transient gene silencing reduced the adhesion of *V. alginolyticus*. (a) qRT‐PCR analysis of the expression of five genes after transient gene silencing at 1, 6, 12, and 24 hr compared with the control. The data are presented as the means ± *SD*, each treatment consisted of six independent biological replicates (3 technical replicates within each). The means of treatments not sharing a common letter are significantly different at *p *<* *.05. (b) The adhesion capacity to mucus of transient silenced *V. alginolyticus* at 2 hr. The data are presented as the means ± *SD*, three independent biological replicates (3 technical replicates within each) were performed per group

The expression levels of these target genes were significantly reduced at 1 and 6 hr after *V. alginolyticus* was treated with siRNAs (Figure 2a). At 1 and 6 hr, transient gene silencing significantly reduced the expression of *secA* (by 2.69‐ and 1.61‐fold, respectively), *secD* (by 8.61‐ and 5.38‐fold, respectively), *secF* (by 3.13‐ and 1.56‐fold, respectively), *yajC* (by 2.16‐ and 1.33‐fold, respectively), and *yidC* (by 4.51‐ and 1.69‐fold, respectively). Reductions in target gene expression indicated that the successful application of these siRNAs.

Since *V. alginolyticus* with RNAi treatments displayed significant silencing at 1–6 hr, the in vitro adhesion assay was performed after transient gene silencing for 2 hr. The results of the in vitro adhesion assay exhibited significantly decreased *V. alginolyticus* adhesion ability under RNAi conditions (Figure 2b). The number of adherent cells of control group was 1349 ± 76 cells/view, whereas in the *secA*‐, *secD*‐, *secF*‐, *yajC*‐, and *yidC*‐RNAi groups, the number were 424 ± 41, 525 ± 34, 312 ± 44, 338 ± 32, and 283 ± 50 cells/view, respectively.

### 3.3 Effects of stable gene silencing

**Figure 3:**
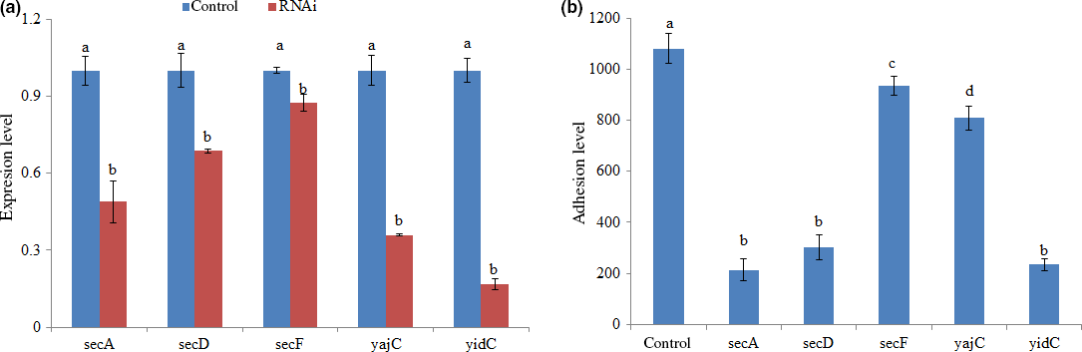
Stable gene silencing reduced the adhesion of *V. alginolyticus*. (a) qRT‐PCR analysis of the expression of *secA*,* secD*,* secF*,* yajC,* and *yidC* after stable gene silencing compared with the control. The data are presented as the means ± *SD*, six independent biological replicates (3 technical replicates within each) were performed per group. The means of treatments not sharing a common letter are significantly different at *p *<* *.05. (b) The adhesion capacity of stable silenced *V. alginolyticus* to mucus. The data are presented as the means ± *SD*, three independent biological replicates (3 technical replicates within each) were performed per group

As shown in Figure 3a, the expression levels of *secA*‐, *secD*‐, *secF*‐, *yajC*‐, and *yidC* were significantly reduced in stably silenced clones by 2.04‐fold, 1.46‐fold, 1.14‐fold, 2.78‐fold, and 5.95‐fold, respectively. These data indicate that the stable gene silencing was reliable in this study.

The stably silenced *V. alginolyticus* exhibited a significant decrease in adhesion. The numbers of adherent bacteria of the control group were approximately 1078 ± 58 cells/view, whereas the corresponding numbers of adherent bacteria of the *secA*‐, *secD*‐, *secF*‐, *yajC*‐, and yidC‐RNAi strains were 214 ± 44, 302 ± 48,932 ± 144, 808 ± 48, and 234 ± 22 cells/view, respectively (Figure 3b). This demonstrated that the adhesion ability of V. alginolyticus was significantly impaired after stable gene silencing. Although *secA*‐RNAi strain exhibited lowest adhesion ability, through synthetical consideration on the gene expression and adhesion after gene silencing, *yajC*‐RNAi seemed to have the smallest effects on adhesion ability.

### 3.4 Effects of different environmental conditions on the gene expression

The expression levels of target genes were affected by the fluctuation of different environmental conditions with different modes.

**Figure 4:**
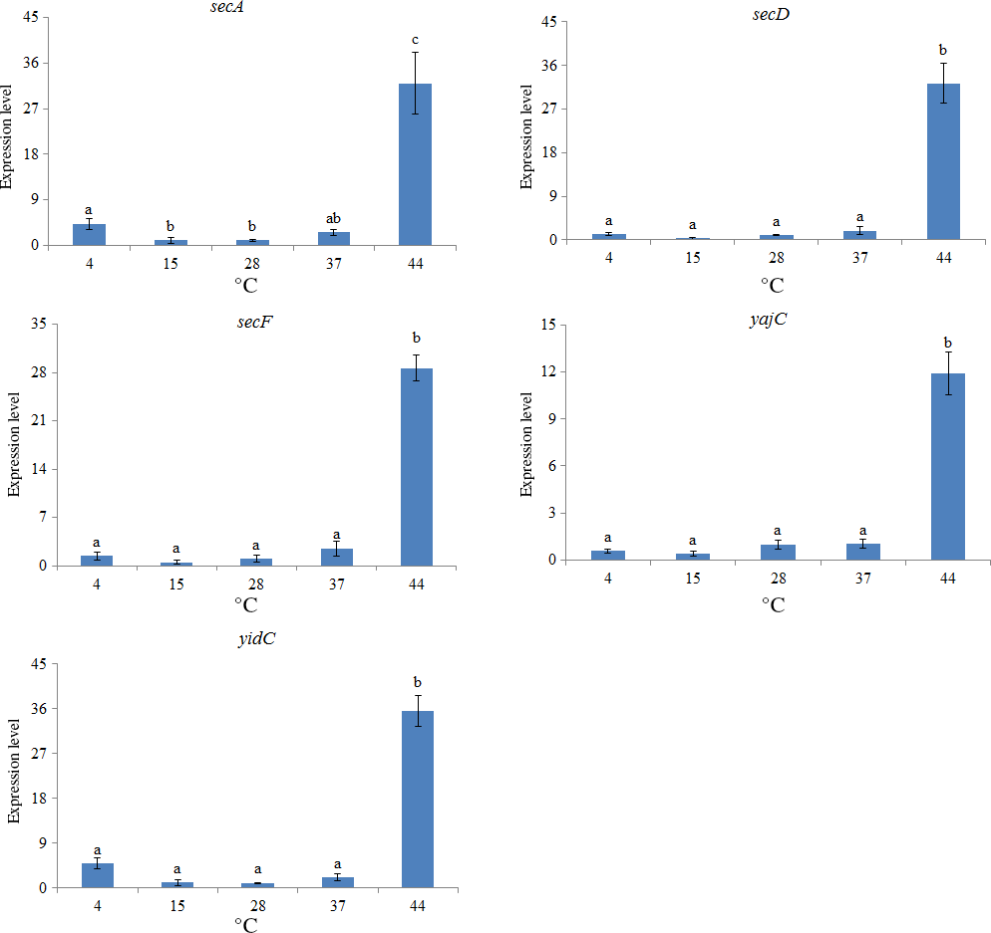
qRT‐PCR analysis of the expression of *secA*,* secD*,* secF*,* yajC,* and *yidC* in the *V. alginolyticus* under different temperatures. The data are presented as the means ± *SD*, each treatment consisted of six independent biological replicates (3 technical replicates within each). The means of treatments not sharing a common letter are significantly different at *p *<* *.05

Temperature exhibited significantly effect on the expression of all the five target genes. The expression of all of the five target genes at 44°C was significantly higher than at other temperatures (Figure 4). Based on the results, high temperatures apparently had a greater impact than low temperatures, and the *yidC* gene appeared to be the most sensitive to high temperature, whereas *yajC* appeared to be the least sensitive to high temperature.

**Figure 5:**
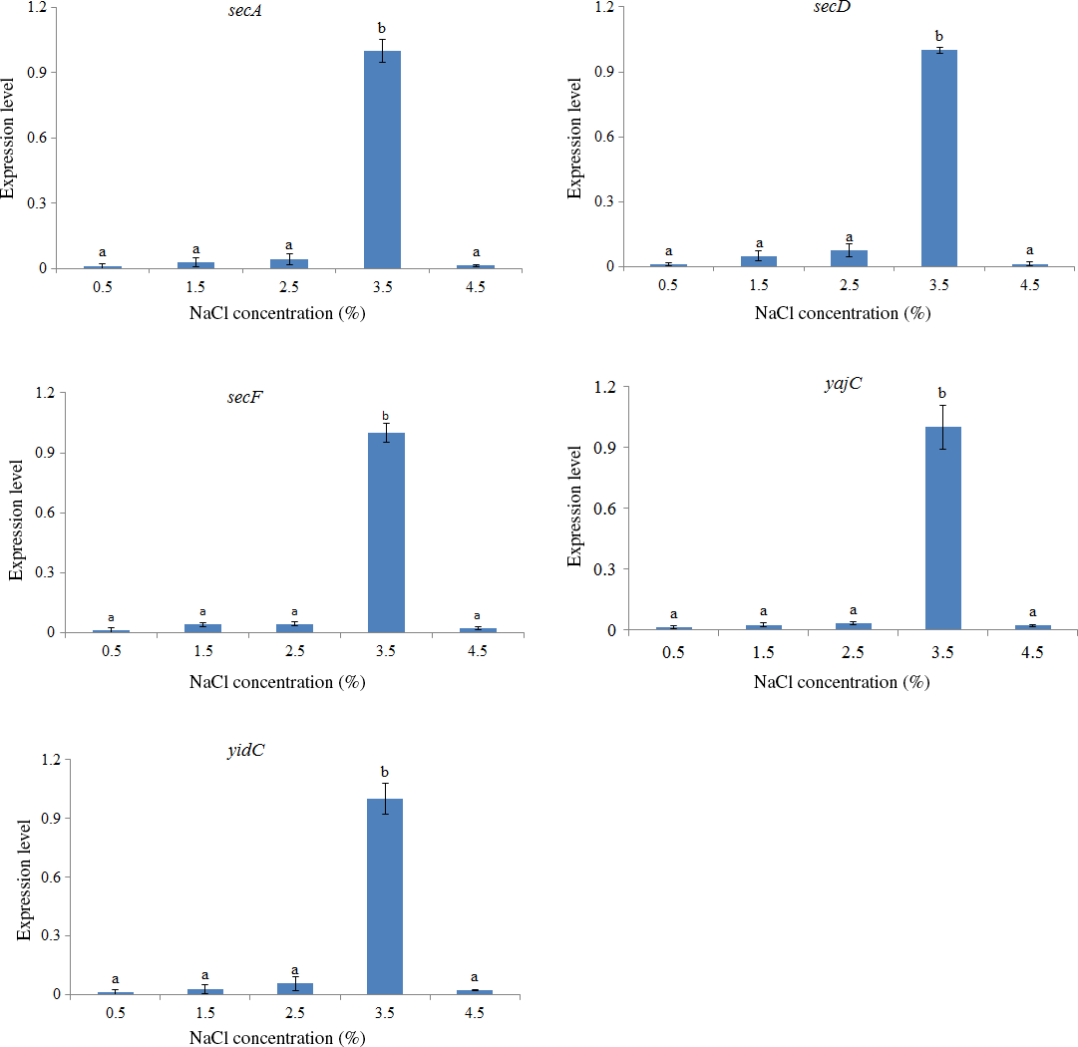
qRT‐PCR analysis of the expression of *secA*,* secD*,* secF*,* yajC,* and *yidC* in the *V. alginolyticus* under different NaCl concentration (%). The data are presented as the means ± *SD*, each treatment consisted of six independent biological replicates (3 technical replicates within each). The means of treatments not sharing a common letter are significantly different at *p *<* *.05

In order to evaluate the response of these genes to salinity changes, the effects of salinity on gene expression were detected and found to be quite similar (Figure 5). The expression of all of the five target genes under NaCl concentration 3.5% was significantly higher than under any other NaCl concentrations. Meanwhile, no significant difference of the expression of the five target genes under other NaCl concentrations was observed (Figure 5).

**Figure 6:**
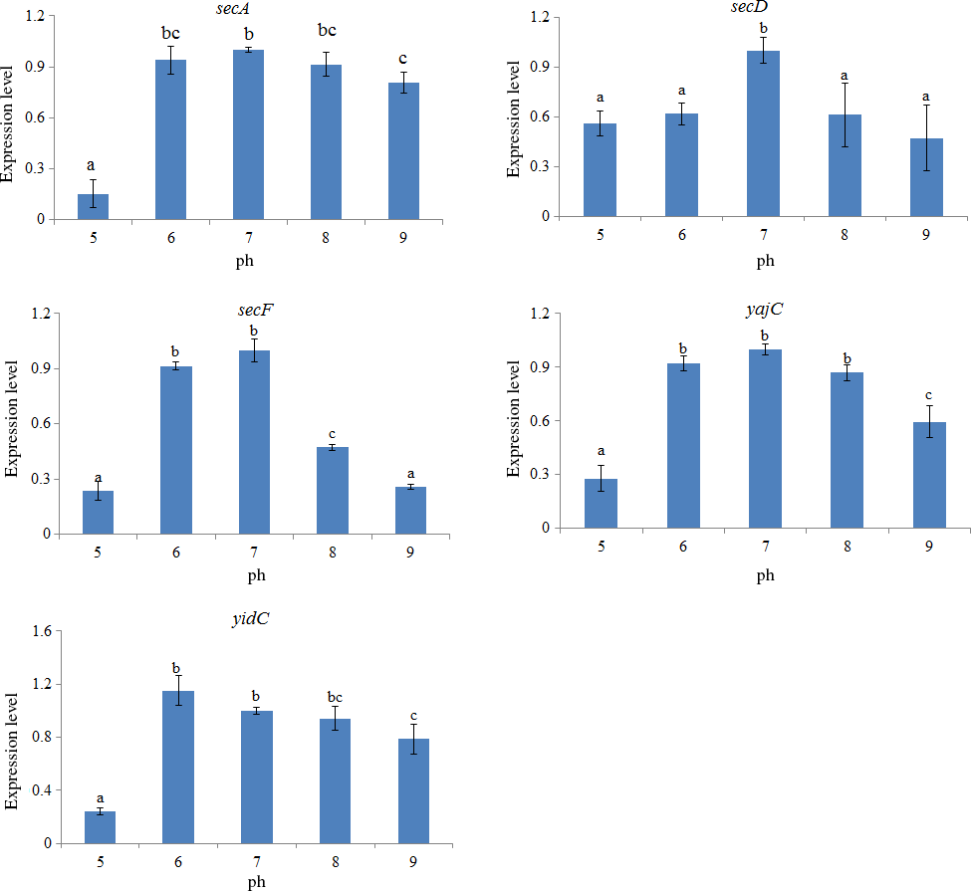
qRT‐PCR analysis of the expression of *secA*,* secD*,* secF*,* yajC,* and *yidC* in the *V. alginolyticus* under various pH values. The data are presented as the means ± *SD*, each treatment consisted of six independent biological replicates (3 technical replicates within each). The means of treatments not sharing a common letter are significantly different at *p *<* *.05

In order to evaluate the response of these genes to pH changes, the expression of the genes was also assessed under different pH levels. The expression levels of the target genes displayed a similar inverted U‐shaped trend (Figure 6). The highest expression was observed at pH = 7, whereas the lowest expression was observed at pH = 5. The *secF* gene appeared to be the most sensitive to different pH, whereas *secA* appeared to be the least sensitive.

**Figure 7:**
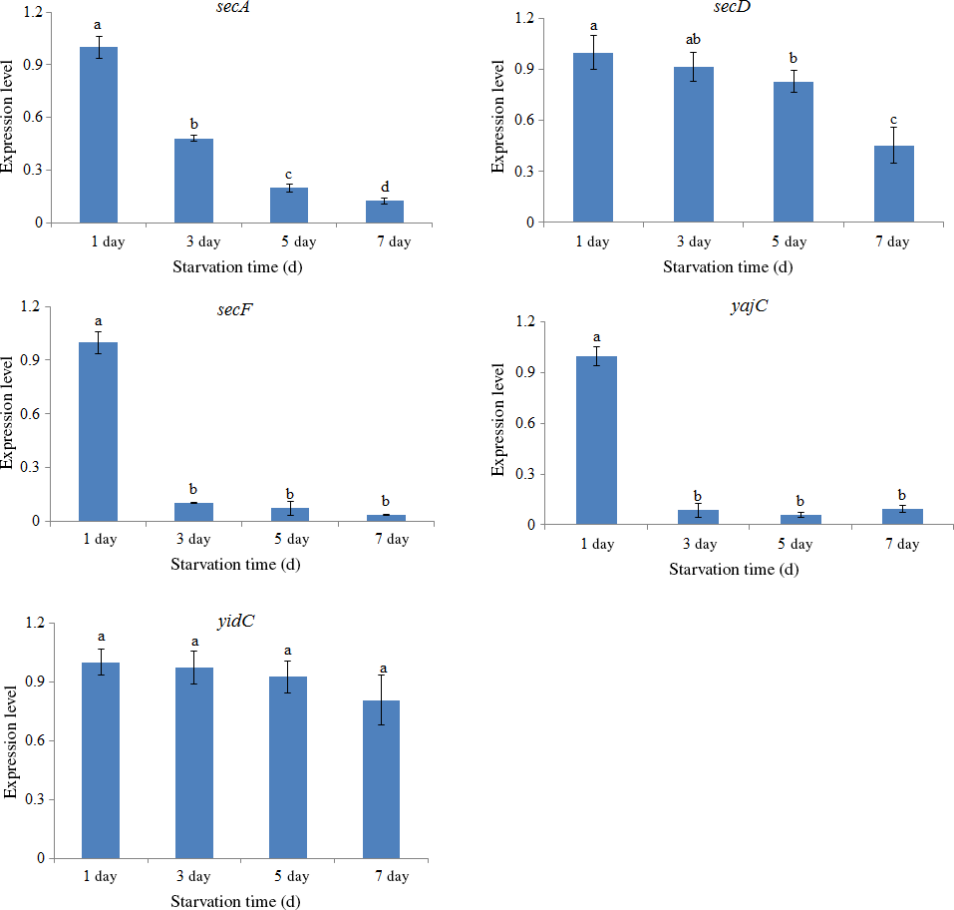
qRT‐PCR analysis of the expression of *secA*,* secD*,* secF*,* yajC,* and *yidC* in the *V. alginolyticus* under different starvation time. The data are presented as the means ± *SD*, each treatment consisted of six independent biological replicates (3 technical replicates within each). The means of treatments not sharing a common letter are significantly different at *p *<* *.05

In order to evaluate the response of these genes to starvation, the expression of the genes was also assessed under starvation. Starvation significantly reduced gene expression in a time‐dependent manner in *secA* since the 3rd day. Starvation also significantly reduced gene expression in *secF* and *yajC* since the 3rd day, but not in a time‐dependent mode. *secD* expression was not affected by starvation until the 5th day. However, starvation exhibited no significantly effect on *yidC* (Figure 7). Furthermore, the expression of *secF* and *yajC* presented a sharp decrease after 3 days of starvation, whereas other three genes did not show such decreases, so it is more like that *secF* and *yajC* were most sensitive to starvation.

## 4 DISCUSSION

The pathogenic process of bacteria is generally divided into adhesion, invasion, colonization, proliferation, and production of toxins (Yan et al., 2007). Bacterial adhesion to host surfaces is one of the initial steps in the infection process (Chen et al., 2008). Host mucus is abundantly found on the surface of the skin, gills, and gut lining; therefore, it is the first site of interaction between the pathogen and its host (Yan, Zhao, Wang, Zou, & Chen, 2010). Several genes have been demonstrated to be involved into the bacterial adhesion to fish mucus (Qin, Yan, Su, Li, & Zou, 2013; Qin et al., 2014; Qin, Lin, Chen, Xu, & Yan, 2016; Huang, Hu, et al., 2016; Huang et al., 2017; Lin et al., 2017). Previous researches on other bacteria have also shown that some secretion systems are necessary for adhesion, mainly through regulating the secretion of adhesin. For example, type V secretion system is involved in the secretion of adhesins such as Trimeric ATs in *Acinetobacter baumannii* (Bentancor, Camacho‐Peiro, Bozkurt‐Guzel, Pier, & Maira‐Litran, 2012); BapA, the chief adhesin of *Paracoccus denitrificans*, was secreted through the type I secretion system (Yoshida, Toyofuku, Obana, & Nomura, 2017); the FhaB/FhaC two‐partner secretion system is involved in fibronectin‐mediated adherence of the *A. baumannii* AbH12O‐A2 isolate (Pérez et al., 2016). Although a combination of structural, biochemical, imaging, computational, and in vivo approaches had led to a working model for the T2SS (Thomassin, Santos Moreno, Guilvout, Tran Van Nhieu, & Francetic, 2017), the role of T2SS played in bacterial adhesion is never concerned before. In this study, RNAi‐mediated silencing of *secA*,* secD*,* secF*,* yajC,* and *yidC* reduced bacterial adhesion to mucus. For the first time, these results demonstrate that T2SS play a key role in *V. alginolyticus* adhesion to mucus. Meanwhile, through synthetical consideration on the gene expression and adhesion after gene silencing, *yajC*‐RNAi seemed to have the smallest effects on adhesion ability.

Although this study proves that *secA*,* secD*,* secF*,* yajC,* and *yidC* play a role in *V. alginolyticus* adhesion, whether it is through the secretion of adhesin and what adhesin was secreted by T2SS is still unclear. Therefore, this study offers new insights, but also raises new questions.

Many pathogenic bacteria can induce an adaptable response to environmental stimuli, primarily by altering gene expression (Bystritskaya et al., 2016). Recent study showed that T2SS promotes not only pathogenic potency, but also environmental survival (von Tils, Blädel, Schmidt, & Heusipp, 2012). However, previous study only concerned about the relationship between temperature and T2SS expression. For example, it is reported that *secD*,* secF,* and *yajC* were linked to sensitivity to growth temperatures (Hand et al., 2006; Liu et al., 2016). The response of T2SS to other environmental factors is never concerned before. In *V. alginolyticus*, it was demonstrated the expression levels of many genes change with environmental factors (Huang et al., 2015; Wang et al., 2015). In this study, the expression levels of *secA*,* secD*,* secF*,* yajC* and *yidC* were found to be sensitive to different temperatures, changes in pH, and increased starvation time. On the other hand, Yan et al. (2007) proved that *V. alginolyticus* adhesion was remarkably influenced by those environmental factors, which suggested that the changes in the expression of *secA*,* secD*,* secF*,* yajC,* and *yidC* may be an important factor influencing adhesion under those environmental conditions. And, for the first time, this study proved that T2SS can response to other environmental factors besides temperature.

Zielke et al., (2014) revealed that the lower temperature had a stimulatory effect on T2SS transcriptional activity in *Vibrio cholera*. Similarly, transcription of T2SS genes was induced in *Yersinia enterocolitica* cultured at 26°C in comparison to those cultured at 37°C (Shutinoski, Schmidt, & Heusipp, 2010), whereas in enterotoxigenic *E. coli*, the expression of the T2SS gene cluster was repressed at lower temperature (Yang, Baldi, Tauschek, Strugnell, & Robins‐Browne, 2007). Considering the recently revealed phylogenetic differences between the T2SS secretins, it is possible that there is a broader bifurcation of T2SS gene expression control across bacterial species (Dunstan et al., 2013). In this study, although *secD*,* secF,* and *yajC* were significantly high expressed at 44°C, no significant difference was found among other growth temperatures. Simultaneously, *secD*,* secF,* and *yajC* did not show more sensitivity to temperatures than *secA* and *yidC*. Therefore, it seems that in *V. alginolyticus*, these genes are sensitive to high temperature, which may be a kind of self‐protection system in adversity.

The expression of *secA*,* secD*,* secF*,* yajC,* and *yidC* at NaCl concentration 3.5% were significantly higher than under other NaCl concentrations, it may be due to *V. alginolyticus* being marine bacteria, which adapted to the marine hypersaline environment.

The expression of *secA*,* secF,* and *yajC* was significant decreased under starvation conditions. The bacterial adhesion of *V. alginolyticus* has been demonstrated to be significant depressed under starvation conditions (Yi et al., 2008). The depression of gene expression of *secA*,* secD*,* secF,* and *yajC* may be one of the reasons why bacteria adhesion is low under starvation conditions. In a nutrient‐poor environment, it might be more cost‐effective to delay energetically expensive secretion until a high density of the bacterial community is reached. Therefore, the low expression of these genes under starvation may be a kind of environmental adaption strategy.

In conclusion, our results indicated that (1) *secA*,* secD*,* secF*,* yajC,* and *yidC* contributed in adhesion *V. alginolyticus*; (2) *secA*,* secD*,* secF*,* yajC,* and *yidC* was sensitive to different temperatures, changes in pH, and increased starvation time. In this paper, we present for the first time the relationship of *secA*,* secD*,* secF*,* yajC,* and *yidC* with bacterial adhesion, which help to promote the understanding of the mechanism of bacterial adhesion.

## CONFLICT OF INTEREST

The authors declare that the research was conducted in the absence of any commercial or financial relationships that could be construed as a potential conflict of interest.
