# Supplementary material for: secA, secD, secF, yajC, and yidC contribute to the adhesion regulation of Vibrio alginolyticus
Source: Microbiologyopen. 2017 Oct 23;7(2):e00551. doi: 10.1002/mbo3.551 (PMC5911994; doi:10.1002/mbo3.551)
Supplement: Supplementary file 1 [file MBO3-7-na-s001.docx]

**Table S1 siRNA Sequence**

| **Target gene** | **siRNA for transient gene silence** |
| --- | --- |
| *secA* | F: 5’ GGUUCUAAAUGCGGGUCAATT 3’  R:5’ UUGACCCGCAUUUAGAACCTT 3’ |
| *secD* | F:5’ CCAAUCAACGACAUUGCAATT 3’  R: 5’UUGCAAUGUCGUUGAUUGGTT 3’ |
| *secF* | F: 5’ GCCAGUUUCACAUCAAGUUTT 3’  R:5’ AACUUGAUGUGAAACUGGCTT 3’ |
| *yajC* | F: 5’ GCGAACAAUGAAGUGGUAATT 3’  R:5’ UUACCACUUCAUUGUUCGCTT 3’ |
| *yidC* | F:5’ GCUCAAGGUCACCAAUUUATT 3’  R:5’ UAAAUUGGUGACCUUGAGCTT 3’ |
| Negative control | F: 5'-UUCUCCGAACGUGUCACGUTT-3'  R: 5'-ACGUGACACGUUCGGAGAATT-3' |

**Table S2 Oligonucleotides used in producing shRNA for stable gene silencing**

| **Target** | **shRNA sequence for** **stable gene silence** |
| --- | --- |
| *secA* | F: 5’GATCCGGTTCTAAATGCGGGTCAATTTTCAAGAGAAATTGACCCGCATTTAGAACCTTTTTTGCATG3’  R: CAAAAAAGGTTCTAAATGCGGGTCAATTTCTCTTGAAAATTGACCCGCATTTAGAACCG |
| *secD* | F: 5’GATCCCCAATCAACGACATTGCAATTTTCAAGAGAAATTGCAATGTCGTTGATTGGTTTTTTGCATG3’  R: CAAAAAACCAATCAACGACATTGCAATTTCTCTTGAAAATTGCAATGTCGTTGATTGGG |
| *secF* | F: 5’GATCCGCCAGTTTCACATCAAGTTTTTTCAAGAGAAAAACTTGATGTGAAACTGGCTTTTTTGCATG3’  R: CAAAAAAGCCAGTTTCACATCAAGTTTTTCTCTTGAAAAAACTTGATGTGAAACTGGCG |
| *yajC* | F:5’ GATCCGCGAACAATGAAGTGGTAATTTTCAAGAGAAATTACCACTTCATTGTTCGCTTTTTTGCATG3’  R: CAAAAAAGCGAACAATGAAGTGGTAATTTCTCTTGAAAATTACCACTTCATTGTTCGCG |
| *yidC* | F: 5’GATCCGCTCAAGGTCACCAATTTATTTTCAAGAGAAATAAATTGGTGACCTTGAGCTTTTTTGCATG3’  R: CAAAAAAGCTCAAGGTCACCAATTTATTTCTCTTGAAAATAAATTGGTGACCTTGAGCG |

**Table S3 Primers for qRT-PCR**

| **Gene** | **Primers for qRT-PCR** |
| --- | --- |
| *secA* | F: 5’ATCGTATGGCGAGCCTTATT 3’  R:5'TGACTGTGGTGGAATGTACTCAT 3’ |
| *secD* | F: 5’ GCATTAGTTCAGCGTCAGGGTA 3’  R:5'CAACAGTTCGGCCATCGTTA 3’ |
| *secF* | F: 5' GTTTGACCAAGGCGGTTTAG 3’  R:TTGGGTAACGGCAAAGAAGC 3’ |
| *yajC* | F: 5’ ATGTTTATTTCTCAGGCTCACGC 3’  R:5’TACCCACTAGGCCACCGCTA 3’ |
| *yidC* | F: 5' GACTACGGCTGGTTATGGTTCA 3’  R:5’GCCACCGAGTGGGTTTACTT 3’ |
